# Sex roles, parental care and offspring growth in two contrasting coucal species

**DOI:** 10.1098/rsos.160463

**Published:** 2016-10-05

**Authors:** Wolfgang Goymann, Ignas Safari, Christina Muck, Ingrid Schwabl

**Affiliations:** 1 Max-Planck-Institut für Ornithologie, Abteilung für Verhaltensneurobiologie, Eberhard-Gwinner-Strasse 6a, 82319 Seewiesen, Germany; 2 Coucal Project, PO Box 26, Chimala, Tanzania; 3 Department of Conservation Biology, University of Dodoma, PO Box 338, Dodoma, Tanzania

**Keywords:** centropus, mating system, nestling growth rate, parental care, sex-role reversal, feeding rate

## Abstract

The decision to provide parental care is often associated with trade-offs, because resources allocated to parental care typically cannot be invested in self-maintenance or mating. In most animals, females provide more parental care than males, but the reason for this pattern is still debated in evolutionary ecology. To better understand sex differences in parental care and its consequences, we need to study closely related species where the sexes differ in offspring care. We investigated parental care in relation to offspring growth in two closely related coucal species that fundamentally differ in sex roles and parental care, but live in the same food-rich habitat with a benign climate and have a similar breeding phenology. Incubation patterns differed and uniparental male black coucals fed their offspring two times more often than female and male white-browed coucals combined. Also, white-browed coucals had more ‘off-times’ than male black coucals, during which they perched and preened. However, these differences in parental care were not reflected in offspring growth, probably because white-browed coucals fed their nestlings a larger proportion of frogs than insects. A food-rich habitat with a benign climate may be a necessary, but—perhaps unsurprisingly—is not a sufficient factor for the evolution of uniparental care. In combination with previous results (Goymann *et al*. 2015 *J. Evol. Biol*. **28**, 1335–1353 (doi:10.1111/jeb.12657)), these data suggest that white-browed coucals may cooperate in parental care, because they lack opportunities to become polygamous rather than because both parents were needed to successfully raise all offspring. Our case study supports recent theory suggesting that permissive environmental conditions in combination with a particular life history may induce sexual selection in females. A positive feedback loop among sexual selection, body size and adult sex-ratio may then stabilize reversed sex roles in competition and parental care.

## 1. Introduction

Parental care—i.e. parental behaviour likely to increase the fitness of offspring—is a fundamental life-history trait that shows substantial diversity within and across animal taxa (reviewed by [1,2]). The decision to provide parental care often results in a resource allocation conflict, because time, nutrients and energy invested into offspring typically cannot be used for other processes such as self-maintenance or searching for additional mating partners (reviewed by [1,3,4]). As a consequence of this conflict, the decision to provide parental care can be closely linked to sexual selection [5,6]. Sexual selection predicts a lower degree of parental care in the sex with a higher variance in mating success. Typically, this variance is higher in males because a male can produce far more sperm than necessary to fertilize the limited number of eggs provided by a female. Also, a low confidence in genetic paternity is more common for males than females and as a consequence should reduce the likelihood of males contributing to parental care [5]. Further, the rarer sex is expected to provide less parental care, because it has more opportunities to find additional mating partners than the more abundant sex [6,7]. This argument is supported by recent comparative studies in shorebirds [8], coucals [9] and birds in general ([10], see also the discussion by Székely *et al*. [11]). Finally, environmental conditions influence parental care decisions, because a low or unpredictable food supply or otherwise harsh conditions typically promote parental cooperation and more equal sex roles in parental care ([4,12], but see [10]). Favourable environmental conditions with a high abundance of food and a climate with benign temperatures, on the other hand, may enable a single parent to successfully raise all offspring and free the other parent to look for additional mating partners [13]. To better understand the potential factors that select for differences in parental care, we need to simultaneously quantify parental behaviour and its effects on offspring (*sensu* [14]) in species with clear sex differences in offspring care. These comparisons gain power if some of the proposed determining factors are controlled for, e.g. by comparing closely related species that breed in the same habitat but differ in parental contributions to offspring care.

Birds are conducive to such studies because they cover the whole range of parental behaviour from almost no care to cooperative breeding. In the majority of bird species (*ca* 81%) both parents provide care, and in cooperative breeders (9%) additional helpers assist in caring. In roughly 8% of all bird species only females care, while male-only care is the exception, occurring in only about 1% of all species [15]. The absence of care (brood parasitism) accounts for the remaining 1% of all birds [15].

In this study, we focused on the exceptional case of male-only care. We compared incubation patterns and nestling feeding rates (as two measures of parental care) with offspring growth (as a measure of parental effects) in two sympatric species of coucals, the biparental white-browed coucal (*Centropus superciliosus*) and the uniparental black coucal (*C. grillii*). These species differ fundamentally in sex roles and mating systems, but otherwise share many life-history traits during breeding (e.g. similar clutch sizes, incubation and nestling periods, and food sources [9]). The aim of this study was to evaluate whether differences in the apparent requirement for parental care or in the growth pattern of young could potentially drive or enhance the differences in sex roles and mating systems between the two species.

Coucals are Old-World non-parasitic cuckoos [16,17] and most of them live in socially monogamous pairs that provide biparental care. Female coucals are usually slightly larger than males and the scarce information that is available suggests that, in general, males contribute slightly more to incubation and offspring care than females [13,18]. We investigated two coucal species that have been described as sister clades ([19], but see [20]) and represent the taxon's two extremes with regard to sexual size dimorphism, mating system and parental care. The white-browed coucal is the least sexually dimorphic of all coucal species (females are around 13% larger than males), and the one with the highest similarity in sex roles. White-browed coucals are socially monogamous, and both partners contribute similarly to territory defence and parental care [9,13,21,22]. With 1.07 males per female, the adult sex-ratio of our breeding population of this species is relatively unbiased [9]. By contrast, the black coucal shows the largest sexual dimorphism of the taxon (females are around 70% larger than males) and experiences the largest difference in sex roles. It has a classically polyandrous mating system, in which females sing and defend large territories, and form polyandrous groups with up to five males, simultaneously. Males incubate their respective clutches and raise the young all by themselves. With 2.49 males per female, the adult sex-ratio of our breeding population of black coucals is strongly male-biased [9,23,24]. At our study site in the Usangu plains, Tanzania, we took advantage of the ideal situation that—during the rainy season starting in December—both species share the same habitat, feed on the same kind of prey and often breed in close proximity to one another.

Seasonal tropical wetlands, such as the Usangu plains, represent the ecosystem type with the highest net primary productivity of any ecosystem (up to 1000 g Cm^−2^ yr^−1^, [25]). During the rainy season, the Usangu plain is partially flooded, leading to a large abundance of insects and small vertebrates such as frogs. Birds of up to 450 species exploit ‘the massive food potential released by the initial flooding of the grasslands’ [26], and temperatures are typically in the range between 20°C and 30°C. Because black coucals are migratory they start breeding later (typically around mid-February [9,23]) than white-browed coucals (typically around early January [9]). When the breeding season ends (around mid-June), most of the black coucals leave the area, whereas the white-browed coucals stay in the Usangu year-round. Hence, their breeding population is limited by the low food availability during the harshest time of the year, the dry season during which the Usangu is desert-like. As a consequence, the breeding density of white-browed coucals is lower than that of black coucals [9].

We have previously [9] identified several factors that may have driven the differences in mating system between the black coucal and other coucal species. These factors were (1) migration and the resulting possibility to exploit seasonally available food resources, thus enabling a high breeding density in black coucals, which facilitated the monopolization of more than one breeding partner, (2) a higher nest predation rate in black coucals with the consequence of (3) female emancipation from incubation and the (4) resulting possibility for females to acquire additional mates. This created a new (5) sexual selection pressure for females to compete for additional mates. As a secondary consequence of factors 1–4, sexual selection may have favoured (6) large females, that are better in competing over territories and mates and that can lay a larger number of eggs, and small males, that are more efficient in providing parental care (see [27] for evidence in raptors). Large body size in females may have promoted reproductive success, but at the same time may have resulted in higher female mortality, leading to (7) a male-biased adult sex-ratio. Sexual selection on large female and small male body size and a male-biased adult sex-ratio probably generated a positive feedback loop stabilizing the mating system. The importance of mutual feedback loops among sexual selection, body size and adult sex-ratio has been pointed out by the seminal work of Andersson [28,29].

Here, we investigated if the requirements for parental care differed between the species and thus could represent an additional factor in maintaining the differences in sex roles between the species. We did so by studying incubation patterns, parental feeding rates and by measuring and comparing ‘off-times’, i.e. the periods of time parenting individuals spent resting and preening on top of grasses or bushes. We hypothesized that if raising a similar number of offspring in white-browed coucals is more costly (i.e. requires more resources or energy) than in black coucals, then a single parent may not be sufficient to raise a clutch. Hence, the larger required effort for offspring care may have stabilized social monogamy and biparental care in white-browed coucals. If this was the case, we would expect that feeding effort and/or offspring growth were substantially higher in biparental white-browed coucals than in uniparental black coucals. If, on the other hand, raising a similar number of offspring in white-browed coucals implies similar costs as in black coucals, then a single parent should be sufficient to raise the young in both species. As a consequence of this, parental care should be less important in contributing to differences in mating system and sex roles between the two species.

Because only males incubate in black coucals [24], but both sexes contribute to incubation in white-browed coucals, we predicted that incubation was more often interrupted in black coucals than in white-browed coucals, and that nests of black coucals were left unattended for longer periods during the day than those of white-browed coucals. If so, this could be one reason why nest predation during the incubation stage is almost twice as high in black coucals (32% of nests) than in white-browed coucals (18%; [9]). Because clutch sizes, number of hatchlings and fledglings are similar in the two species, but nestlings of white-browed coucals leave the nest slightly later and at a larger body mass than black coucals [9] we expected overall higher feeding rates in white-browed coucals, even though each of the two parents may contribute less than a single uniparental male black coucal. As a consequence, we also predicted that female and male white-browed coucals would spend more time resting on grasses or bushes than male black coucals. Female black coucals do not provide parental care, but mainly use perches as song posts and to overlook their territories [23] and hence were predicted to spend more time on bushes or grasses than conspecific males or white-browed coucals. We also asked whether potential species differences in parental care resulted in differences in offspring growth. We predicted that an overall higher feeding rate would result in larger parental effects, represented by a higher offspring growth rate in the species with the higher feeding effort.

## 2. Material and methods

We studied a population of black and white-browed coucals in the partially flooded grassland of the Usangu wetland (8°41′ S and 34°5′ E; 1000 m a.s.l.) in Mbeya region, Tanzania, during the breeding seasons of 2005 (23 January–27 May), 2006 (15 January–25 April), 2008 (10 February–13 March), 2010 (20 February–25 April), 2011 (18 February–6 June), 2012 (18 December–18 June), 2013 (25 January–29 June), 2014 (15 January–29 June) and 2015 (3 February–27 June). For further details regarding the study site, see [9].

Adult coucals were caught with mist nets and banded with numbered aluminium and coloured plastic rings for individual identification. During the above-mentioned study years, we caught and ringed a total of 120 adult female and 99 adult male black coucals, as well as 33 adult female and 66 adult male white-browed coucals. Most birds were equipped with a Holohil BD-2 radio-transmitter (less than 2 g; Holohil Systems Ltd., Carp, Ontario, Canada) to ease individual identification and location of individuals (see [9] for more details on capture, measurement and tagging).

Nests of coucals were located by following adult birds carrying nesting material or food in their beaks, or by finding incubating birds that had been equipped with radio-transmitters (as described by Goymann *et al*. [9]). During the incubation stage, nests were checked every fourth day to see whether young had hatched. At the expected end of the incubation, nest checks were conducted more frequently (approx. every 2 days). To study a potential sex bias in incubation patterns, we attempted to identify the individual that left the nest when we approached during these checks. In addition, we conducted incubation checks from a distance using the signals of radio-tagged birds. These regular checks were conducted at various times of the day (07.00–17.00 h).

**Figure 1.**
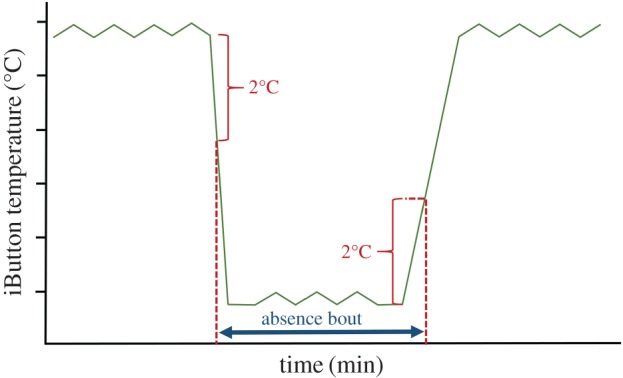
Illustration of the definition of incubation interruptions and the measurement of the duration of such absence bouts during the incubation period. We defined the beginning of an absence bout during incubation as a steady drop in the recorded iButton temperature (green line) of at least 2°C in magnitude (upper left red bracket). The end of the incubation absence bout was defined as the point in time when the iButton temperature had increased by 2°C above the minimum temperature recorded during the absence bout (lower right red bracket). The duration of each absence bout was defined as the time period in between these two defined time points (blue bar with double arrows).

To study nest attendance during incubation, in 2005 and 2006 we placed temperature loggers (Thermochron iButtons, Maxim Integrated Products, Inc. USA) wrapped into leaves underneath the eggs into 10 different black coucal and 11 different white-browed coucal nests. The loggers recorded the nest temperature (in 0.5°C increments) in 3 min intervals and were removed for data extraction after 3–6 days. Because the loggers were placed underneath the eggs the recorded nest temperature did not reflect actual incubation temperatures. However, changes in temperature allowed us to calculate how often incubation was interrupted, the duration of each interruption and the total amount of time birds were absent from the nest each day. We defined the beginning of each interruption of incubation as a steady drop in the recorded temperature of at least 2°C in magnitude. The duration of each absence bout was estimated as the time period during which the nest temperature remained within 2°C of the minimum temperature recorded during the respective absence bout (see figure 1 for an illustration).

Once at least one nestling had hatched, nests were checked and the nestlings measured every other day until they left the nest. To distinguish individual nestlings, we marked two of their four claws of one foot with nail enamel in a unique way, and when they were 9–11 days old they received a numbered aluminium ring. To estimate nestling growth we measured body mass (to the nearest gram), and the length of the right tarsus (to the nearest 0.1 mm) as a measure of structural growth. When the nestlings were around 5–7 days old, we took a small blood sample (approx. 30 µl) in Queens lysis buffer for genetic sexing [30].

To determine parental feeding rates, we conducted focal observations of nests (typically of 60 min duration) using binoculars from a distance of around 40–100 m, sitting on the roof of a car, on top of a ladder or on a mud bank. We identified the individual coming to the nest, scoring a nest visit as a ‘feeding visit’ only when the arriving bird carried food in its bill. Whenever possible, we tried to identify the kind of food brought to the nest. Further, we recorded the time individuals spent visibly perching high on bushes or grasses without food. Because coucals do not hunt from perches (personal observation of all authors), we consider the time they spend perching high as a conservative estimate of ‘off-times’, i.e. the time they do not spend searching for prey, but rest and preen. We did not count perching incidents with food in the beak as ‘off-time’ because similar to many other birds coucals wait and carefully scan the environment before approaching the nest with food for the nestlings. All procedures were approved by the respective governmental authorities of Tanzania, i.e. the Tanzania Wildlife Research Institute (TAWIRI) and the Tanzanian Commission for Science and Technology (COSTECH).

All statistical analyses were conducted with R, v. 3.2.2 (R Core Development Team, Vienna 2015). We tested for a potential sex bias in incubation behaviour in white-browed coucals using a generalized linear mixed model (glmer in R package nlme [31]) with a binomial distribution (presence/absence) and a loglink function, with the sex of the incubating bird as dependent variable, the time of day (centred) as a covariate and the nest ID as a random factor. To test whether factors such as Julian date or year of study would influence the results, we initially included them as random effect (year) or covariate (Julian date), but since they did not have an influence we did not include them in the final analysis. The other variables were maintained in the analysis because we expected them to potentially have a meaningful impact. The analysis was based on 154 observations on 28 nests. For black coucals, we did not conduct a statistical analysis of sex bias in incubation patterns, because female incubation was never observed in this species.

The number of absence bouts from the nest (i.e. interruptions of incubation) per day and the total absence duration (in minutes per day) from the nest were compared between the two species using linear mixed models (lmer in R package nlme [31]) with species as fixed factor and nest identity as a random factor. Each nest contributed three to six data points, depending on whether the logger recorded for 3, 4, 5 or 6 days. The mean durations of absence bouts were analysed using a linear mixed model (lmer in R package nlme [31]) with species and time of day (morning (06.00–10.30), noon (10.30–15.00) and afternoon (15.00–19.30)) as independent variables, and nest identity as a random factor. Here, each nest typically contributed three values per day.

The feeding rate analysis was based on 256 individual observations of 76 black coucal nests (302 h), and 184 feeding observations of 54 white-browed coucal nests (209 h). During each observation, we quantified the number of feeding visits by the male and the female. Each nest contributed at least 2 × 60 min of total focal observations (range: 2–7 observations of 30–225 min duration). Feeding rates were statistically analysed using a generalized linear mixed model (glmer in R package nlme [31]) with a Poisson distribution, using the number of feeding visits of an individual as dependent variable and the duration of the observation in minutes as an offset (following the recommendations by Korner-Nievergelt *et al.* [32]) to control for differences in the duration of observations. Independent variables were species, sex, the interaction between species and sex, and the total brood mass (i.e. combined body mass of all nestlings on the respective day of observation), as well as the time of day as covariates. We decided to use brood mass as covariate, because in our view it represents an accurate combined measure of differences in the number and age of nestlings, especially in coucals where nestlings hatch asynchronously, and where brood sizes differ. In the electronic supplementary material, S1, we also present an alternative analysis in which brood mass was replaced with the number of nestlings and mean nestling age as covariates. The covariates were centred and scaled to allow an unbiased estimation of the parameters. The identity of the feeding parent and nest ID were included as random effects. To test whether year of study would influence the results, we initially included it as a random effect, but since it did not explain any random variance in addition to that explained by parent ID and nest ID we did not include year in the final analysis.

The analysis of ‘off-times’, i.e. the time that white-browed coucals and male black coucals spent perching high on grasses or bushes in between feeding visits to the nest, was—similar to the feeding rate analysis—conducted by using a generalized linear mixed model (glmer in R package nlme [31]) assuming a normal distribution, using the time an individual perched on a bush or grass as dependent variable and the duration of the observation in minutes as an offset (following the recommendations by Korner-Nievergelt *et al.* [32]) to control for differences in the duration of observations. Independent variables were species, sex, the interaction between species and sex, and the total brood mass (i.e. combined body mass of all nestlings on the respective day of observation), as well as the time of day as covariates. The covariates were centred and scaled to allow an unbiased estimation of the parameters. The identity of the feeding parent and the nest were included as random effects. In cases where this was possible, the female partners of feeding male black coucals were included in the ‘off-time’ analysis, although female black coucals mainly perched to sing and guard their territories and hence the results on females did not represent real ‘off-times’ [23].

The difference in the proportion of the main food items (frogs, grasshoppers, mantises) brought to the nest was statistically analysed using the bayes.prop.test from the R package BayesianFirstAid [33] and compared between black and white-browed coucals using the *region of practical equivalence* (ROPE; [34]), which can be considered as a measure of the likelihood that two distributions are similar to each other.

Nestling growth rates were estimated for each sex and species separately with a nonlinear logistic function: weight = *A*/[1 + exp(−*K* × {*t* − *I*})] with *A* = asymptote of nestling body mass (in grams) before leaving the nest, *K* = growth rate constant, *I* = inflection point of the growth curve (in days) and *t* = age (in days) [35]. We used a nonlinear mixed model approach (function nlme in R package nlme, [31]) following Sofaer *et al*. [36]. Nestling ID was included as a random effect to model repeated measurements of individuals. The nestling ID was nested within nest ID as another random effect to control for non-independence of nest-mates due to a common nest environment, genetic background, maternal effects and parental care [36]. As the starting values for the estimation of growth parameters, we used the parameter values for *A*, *I* and *K* (for each sex separately) from a previous study on black coucals [24]. For the estimation of growth curves in white-browed coucals, we used the *I* and *K* values of female black coucals as starting values, and we set the initial value of *A* to 92.4 g for females and 89.5 g for males, which corresponds to the mean body mass of females and males shortly before they left the nest [9]. The growth rate analyses were based on data from 118 female (contributing 610 data points) and 122 male (contributing 639 data points) nestlings from 63 black coucal nests, and from 102 female (contributing 566 data points) and 109 male (contributing 598 data points) nestlings from 60 white-browed coucal nests.

Similar analyses were conducted using tarsus length as the dependent variable. Because the results were similar to the body mass analyses, the results for the tarsus data are reported only in the electronic supplementary material, S2.

Model residuals were examined using graphical methods (i.e. qq plots of residuals and random effects, fitted values versus residuals) for homogeneity of variance, violation of normality assumptions or other departures from model assumptions and model fit [32]. For inferences from the models, we obtained Bayesian parameter estimates and their 95% credible intervals (using bsim of the R package arm [37], with an uninformed prior distribution [32]). The Bayesian approach is the only method that allows the drawing of exact inferences while avoiding the difficulties of determining the degrees of freedom in mixed model analyses [38]. Another major difference is that Bayesian methods refer to the probability of a hypothesis given the data. By contrast, frequentist methods assess the probability of the data given a null hypothesis. Hence, unlike null hypothesis testing, Bayesian methods do not provide *p*-values. Instead, biologically meaningful differences between groups can be assessed by comparing the ranges of the 95% credible intervals between groups. Similarly, the 95% credible interval of a regression slope can be used to assess the relationship between continuous variables. The 95% credible interval provides an estimate for the mean with a probability of 0.95. If the credible interval of one group does not overlap with the mean estimate of another group, the groups can be assumed to differ from each other. Similarly, if the 95% credible interval of the slope in a regression does not include zero it can be assumed that there is a meaningful relationship between the continuous variables [32]. If not indicated otherwise, data are presented as individual data points in combination with posterior means and their respective 95% credible intervals (in text and tables reported within squared brackets). We also provide measures of the goodness of fit of the models (i.e. how much of the variance they explain) by reporting *R*^2^-values for linear models, or the respective marginal and conditional *R*^2^-values for mixed models following Nakagawa & Schielzeth [39]. The marginal *R*^2^-value represents the variation explained by the fixed effects of a mixed model, whereas the conditional *R*^2^-value reflects the combined variation explained by fixed and random effects. Because a Bayesian approach was not feasible for the analysis of the growth rate data, we report mean estimates and their 95% confidence intervals for the growth rate analyses, instead.

## 3. Results

### 3.1. Incubation patterns

In none of the 191 black coucal nests found during the incubation stage in the study period did we ever observe a female incubating the clutch, confirming earlier observations that in this species only males incubate [23]. Because of this obligate sex bias in diurnal and nocturnal incubation, a formal binomial statistical comparison between the sexes seemed redundant in black coucals. In white-browed coucals, both females and males incubated. Yet, a binomial mixed model of all cases where we could identify the sex of the incubating bird indicated that in 80.9% [95% credible interval: 65.8–90.2%] of all cases the males were incubating the clutch. The likelihood of male incubation increased from morning to evening (slope 0.64 [0.11–0.99]; *N* = 154 observations of 28 nests; marginal *R*^2^ = 0.088, conditional *R*^2^ = 0.720). The high conditional *R*^2^-value demonstrates that nest ID explained a large proportion of the variance in the white-browed coucal data, suggesting a high consistency in the proportion of male and female incubation per nest. Because we lack information about the identity of individuals incubating at night, we cannot rule out the possibility that female white-browed coucals contribute more to incubation at night.

**Figure 2.**
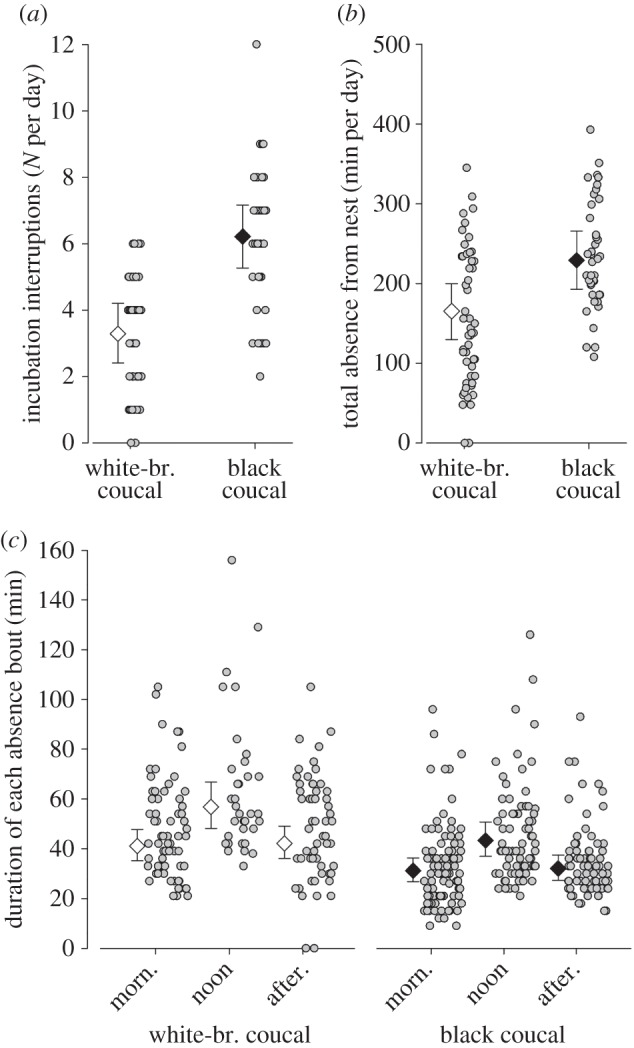
Individual measures (grey dots) and posterior Bayesian estimates with 95% credible intervals of (*a*) the number of daily interruptions from incubation, (*b*) the total duration of all interruptions of incubation per day and (*c*) the duration of each incubation pause of white-browed coucals (large white diamonds) and black coucals (large black diamonds). The analysis is based on nest temperature data for 40 days from 10 different black coucal nests, and 51 days from 11 different white-browed coucal nests. In Bayesian analysis, differences between groups can be considered biologically meaningful, if the 95% credible intervals (whiskers) of one group do not overlap with the mean estimate of another group.

The iButton data indicated that incubation was more frequently interrupted in nests of black coucals than in nests of white-browed coucals (figure 2*a*; marginal *R*^2^ = 0.384, conditional *R*^2^ = 0.690). Also, the total duration nests remained unattended was longer in black coucals than in white-browed coucals (figure 2*b*; marginal *R*^2^ = 0.142, conditional *R*^2^ = 0.451). However, the duration of each absence bout was shorter in black than in white-browed coucals (figure 2*c*; marginal *R*^2^ = 0.119, conditional *R*^2^ = 0.261). Both species spent longer periods away from the nest during the middle of the day, when ambient temperatures were high than during the mornings or afternoons (figure 2*c*).

### 3.2. Nestling feeding rates and food types delivered

**Figure 3.**
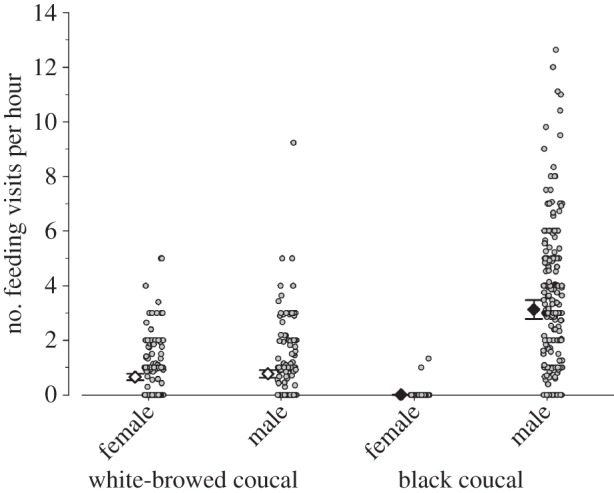
Posterior Bayesian estimates with 95% credible intervals of mean feeding rates of white-browed coucals (large white diamonds) and black coucals (large black diamonds). Small grey dots indicate the respective feeding rate of each individual observation. The analysis is based on 256 individual observations of 76 black coucal nests (302 nest hours), and 184 feeding observations of 54 white-browed coucal nests (209 nest hours). For the statistical interpretation of meaningful differences, see figure 2 and Material and methods.

The offspring feeding rates differed between the two species and sexes (species × sex interaction: *F* = 89.97, species *F* = 203.88, sex *F* = 11.02; figure 3): in black coucals, the young were almost exclusively fed by males (figure 3; with the exception of one female at one nest (2 feeding observations) feeding rates of females were zero at all other 75 nests (254 feeding observations)). In white-browed coucals, females and males fed their offspring at similar rates, but each parent delivered food at a much lower rate than male black coucals (figure 3). In both species, the feeding rate increased with total brood mass (back-transformed slope 0.31 [0.22–0.39], *F* = 57.54), but time of day did not influence feeding rates (back-transformed slope −0.01 [−0.07 to 0.048], *F* = 0.15). The overall model explained a large proportion of the variance in the data (marginal *R*^2^ = 0.827, conditional *R*^2^ = 0.848). Even when the average feeding visits of female and male white-browed coucals were summed for each nest, these visits represented only about half of the feeding visits conducted by a single male black coucal (figure 3). We did not quantify the length of the foraging trips (i.e. distance travelled per trip), but from our observations we did not find obvious differences between the species, and also territory or home-range sizes were similar between male black coucals and both sexes of white-browed coucals [9], suggesting a similar ranging behaviour. A similar model using the number of nestlings and the mean age of all nestlings in a nest rendered very similar results to this model that used the mean brood mass (as a composite measure of the number of nestlings and their age) as a covariate (see the electronic supplementary material, S1).

Black and white-browed coucals mainly delivered grasshoppers (*Caelifera*), frogs (*Anura*) and mantis species (*Mantodea*) to their nestlings. However, the proportion of these three prey types differed between the two species: white-browed coucals delivered a higher percentage of frogs, whereas male black coucals fed a larger proportion of grasshoppers (table 1). An analysis of the regions of practical equivalence (ROPE) indicated that there was only a 0.013% likelihood that black and white-browed coucals fed a similar proportion of frogs (ROPE = 0.00013), and only a 3.2% likelihood that they fed a similar proportion of grasshoppers (ROPE = 0.032). By contrast, the likelihood that the two species fed a similar proportion of mantises was relatively high (43%; ROPE = 0.43).

**Table 1.** Number of grasshoppers, frogs and mantises brought to the nest by black coucals (*N* = 76 nests) and white-browed coucals (*N* = 54 nests). In brackets are the posterior mean proportions of the respective prey items with their Bayesian credible intervals. The lack of overlap between the credible intervals and the mean proportion of grasshoppers and frogs fed by black and white-browed coucals indicated that the two species differed in how much of these prey items they delivered to their nestlings.

species	grasshoppers (*Caelifera*)	frogs (*Anura*)	mantises (*Mantodea*)
black coucal	489 (0.73 [0.69–0.76])	96 (0.14 [0.12–0.17])	88 (0.13 [0.11–0.16])
white-browed coucal	197 (0.62 [0.57–0.67])	94 (0.30 [0.25–0.34])	25 (0.08 [0.05–0.11])

### 3.3. Off-times

**Figure 4.**
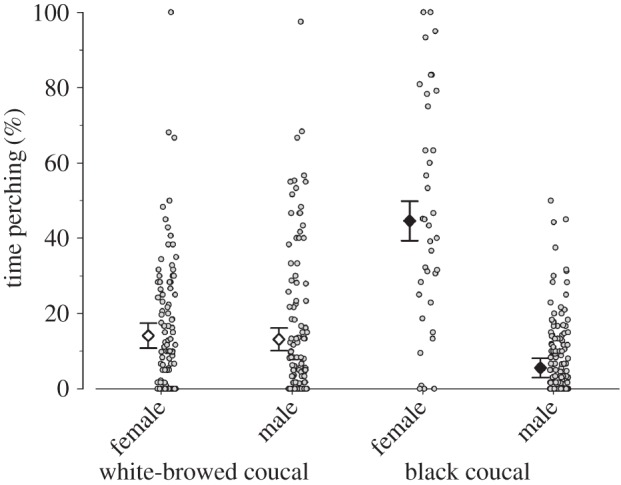
Posterior Bayesian estimates with 95% credible intervals of the mean proportion of time female and male white-browed coucals (large white diamonds) and male black coucals (large black diamonds) spent resting on grasses or bushes when they had dependent offspring. For female black coucals, these ‘off-times’ rather represent times they spent perching high to sing and guard their territory. Small grey dots indicate the respective proportion of time a bird spent resting for each individual observation. For sample sizes, see figure 3 and for the statistical interpretation of meaningful differences see figure 2 and Material and methods.

The time periods individuals spent perching high on bushes or grasses differed between the two species and sexes (species × sex interaction: *F* = 118.15, species *F* = 1.29, sex *F* = 69.11; figure 4): feeding male black coucals spent little time perching high on grasses or bushes. When they did so, they typically preened or dried their plumage in the sun. By contrast, female black coucals spent considerable time perching high on grasses or bushes. Female black coucals also preened themselves while perching high, but they frequently used the perches also as song posts. In white-browed coucals, feeding females and feeding males spent similar times perching on grasses or bushes, and they spent more time doing so than feeding male black coucals (figure 4). Also white-browed coucals typically preened themselves and dried their plumage in the sun when perching high. In both species, the time spent perching high on grasses and bushes did not depend on total brood mass (slope 0.006 [−0.008 to 0.022], *F* = 0.76), but decreased during the day (slope −0.021 [−0.036 to −0.008], *F* = −3.07). The overall model explained about a third of the variance in the data (marginal *R*^2^ = 0.290, conditional *R*^2^ = 0.344).

### 3.4. Growth rates

**Figure 5.**
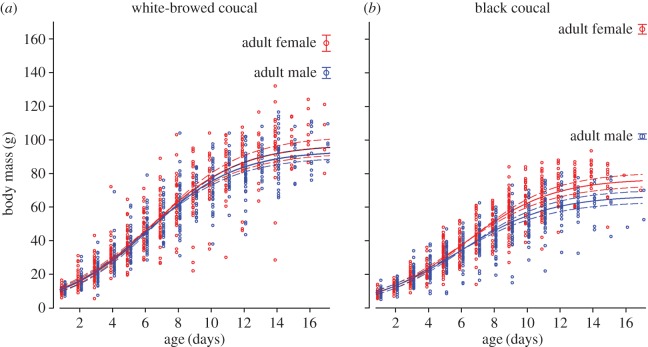
Growth rate of female (in red) and male (in blue) white-browed coucals (*a*) and female and male black coucals (*b*). Solid and dashed curves represent the mean growth curve and their respective 95% confidence intervals. Individual dots refer to data points from individual nestlings. The asymptotes of the growth curves indicate that white-browed coucals reached a higher asymptotic body mass than black coucals before leaving the nest. White-browed coucals and male black coucals left the nest at similar body masses relative to the adult body mass (indicated in the graphs: female white-browed coucals: 63.2%; male white-browed coucals: 68.7%; male black coucals: 68.3%), whereas female black coucals left the nest at a considerably lower body mass relative to the adult body mass (46.5%). In black coucals, the number of individual body mass data points in the nest drops after day 13, a similar drop in body mass data occurs only after day 14 in white browed coucals, indicating that black coucals typically leave the nest slightly earlier than white-browed coucals (for a formal analysis, see [9]).

On the species level, the growth constants *K* and their 95% confidence intervals indicated that black and white-browed coucals grew at similar rates with little difference between the sexes (table 2). However, male black coucals reached the period of maximum growth (inflection point I) earlier than females, and they also reached the inflection point earlier than both sexes of white-browed coucals (figure 5 and table 2). The asymptotic body mass of the young just before leaving the nest (around days 13–16) also differed between the species and the sexes. Female black coucals reached a higher asymptotic body mass than males, and both female and male white-browed coucals reached a higher asymptotic body mass than female and male black coucals (figure 5 and table 2). Black coucals typically left the nest at an earlier age than white-browed coucals (see legend of figure 5 and [9]). Female black coucals left the nest at the lowest body mass relative to their adult body mass. Their asymptotic body mass when leaving the nest corresponded to 46.5% of the typical adult body mass of a female black coucal (165.9 g), whereas males left the nest at 68.3% of adult body mass (98.0 g), which is similar to the relative adult body mass of white-browed coucals leaving the nest (63.2% in females (153.8 g) and 68.7% in males (136.1 g); see table 2 for asymptotic body masses when leaving the nest and figure 5 and [9] for a representation of adult body masses).

**Table 2.** Mean estimates and 95% confidence intervals for the growth constant (*K*), the inflection point (*I*) and the asymptotic body mass before leaving the nest (*A*) for white-browed and black coucals. The analyses were based on data from 118 female (contributing 610 data points) and 122 male (contributing 639 data points) nestlings from 63 black coucal nests, and from 102 female (contributing 566 data points) and 109 male (contributing 598 data points) nestlings from 60 white-browed coucal nests.

species	sex	growth constant (*K*)	inflection point *I* (day)	asymptotic body mass *A* (g)
white-browed coucal	female	0.369 [0.354–0.384]	6.3 [6.0–6.6]	97.2 [92.3–102.1]
		*t*_462_ = 46.95	*t*_462_ = 39.76	*t*_462_ = 39.02
	male	0.375[0.362–0.388]	6.2 [6.0–6.5]	93.5 [90.0–97.0]
		*t*_487_ = 55.36	*t*_487_ = 44.18	*t*_487_ = 52.01
black coucal	female	0.377 [0.363–0.392]	6.2 [5.9–6.5]	77.1 [73.2–80.9]
		*t*_490_ = 51.01	*t*_490_ = 40.00	*t*_490_ = 39.27
	male	0.364 [0.347–0.381]	5.8 [5.5–6.2]	66.9 [63.4–70.5]
		*t*_515_ = 41.35	*t*_515_ = 34.35	*t*_515_ = 37.24

On the level of individual nests, there were consistent correlations between asymptotic body mass and the inflection point in black coucals: in nests where nestlings reached the inflection point earlier the nestlings also reached a higher asymptotic body mass (as indicated by the fact that the 95% confidence intervals of the correlation coefficient did not include zero; table 3). Thus, the early growth pattern of all nestlings of a nest corresponded with the asymptotic body mass that they reached (i.e. the earlier the inflection point, the higher the asymptotic body mass). In white-browed coucals, this was not the case, because the 95% confidence intervals of the correlation coefficients included zero (table 3).

**Table 3.** Estimates and their 95% confidence intervals for the standard deviations of random effects of asymptotic body mass before leaving the nest (*A*) and the inflection point (*I*), and their respective correlations (with 95% confidence intervals) on the nest (left) and the nestling level (right). Biologically meaningful correlation coefficients are highlighted in italics.

		nest ID			nestling ID		
species	sex	asymptote *A*	infl. point *I*	correlation	asymptote *A*	infl. point *I*	correlation
black coucal	female	9.3 [5.9–14.6]	0.9 [0.7 –1.2]	*0*.*8* [*0*.*4*–*0*.*9*]	11.7 [9.2 –15.0]	0.4 [0.2 –0.7]	0.4 [−0.1–0.8]
	male	10.8 [8.4 –13.8]	0.9 [0.7 –1.2]	*0*.*7* [*0*.*4*–*0*.*9*]	5.6 [4.0 –7.8]	0.7 [0.5 –0.9]	−0.2 [−0.6 –0.4]
white-browed coucal	female	11.4 [7.3–17.8]	0.6 [0.2–2.0]	0.4 [−0.4–0.9]	14.5 [11.2–18.9]	1.0 [0.7–1.5]	*0*.*6* [*0*.*3–0*.*8*]
	male	7.7 [4.8–12.4]	0.8 [0.6–1.1]	0.2 [−0.4–0.6]	10.8 [8.6–13.6]	0.6 [0.4–0.8]	*0*.*5* [*0*.*1–0*.*8*]

On the level of individual nestlings, inflection points and asymptotic body masses showed consistent correlations in both sexes of white-browed coucals, but not in black coucals (table 3, right column). This suggested that the time of maximum growth and the asymptotic body mass at the end of the nestling period were strongly connected in individual nestlings of white-browed coucals, but not in black coucals. Overall, individual nests and individual nestlings varied in asymptotic body masses and inflection points, as indicated in the estimated random effects standard deviations and their respective 95% confidence intervals (table 3). This strong influence of the random effects suggested that consistent differences on the nest level and individual nestling level played a role in growth parameter estimation (for additional results regarding growth parameter estimates of tarsus length, see the electronic supplementary material, S2).

## 4. Discussion

In this study, we compared parental care and offspring growth (parental effects) between biparental white-browed and uniparental black coucals. The degree of parental care was considerably smaller in individual female and male white-browed coucals than in individual male black coucals. As predicted, male black coucals interrupted incubation more often than white-browed coucals, and even though these interruptions were shorter, black coucals spent more time away from the nest during the day than white-browed coucals. In contrast to our predictions, male black coucals conducted substantially more feeding visits: they fed their young more than twice as often as breeding pairs of white-browed coucals. Further, male black coucals spent less than half of the time with resting and preening than white-browed coucals. With one exception, female black coucals did not provision the young during the course of this study (see also [23] for a similar case). Hence, parental care was substantially higher on a per-parent basis in male black coucals, because individual males fed their offspring around four to five times more often than individual members of a white-browed coucal pair. Further, parental care was substantially higher in male black coucals also on an overall (per nest) basis, because uniparental male black coucals provisioned their offspring more than twice as often as biparental white-browed coucals. Female black coucals spent a large proportion of time on small bushes or high grasses, which is typical because they use elevated perches as song posts and to guard their territories.

The larger investment in parental care of male black coucals did not result in higher offspring growth because important body mass growth parameters—such as the growth constant *K*, and the inflection point *I*—did not differ between the two species. By contrast, nestlings of white-browed coucals reached a larger asymptotic body mass before they left the nest (but they also left the nest at an older age, see [9]). Relative to their adult body masses, female and male white-browed coucals and male black coucals left the nests at a similar body mass, but female black coucals left the nest at a much lower body mass relative to their typical adult body mass. Hence, female black coucals are likely to have a higher risk of juvenile mortality than conspecific males or both sexes of white-browed coucals [9].

Because the number of nestlings is similar in the two species [9] the feeding and growth data raise the question as to how white-browed coucal nestlings could maintain a similar growth as black coucals, despite the fact that they were fed less frequently? Adult white-browed coucals may have compensated the fewer feeding trips by feeding a larger proportion of frogs than male black coucals. Such frogs represent relatively large prey and one frog could even be split between nestlings. By contrast, black coucals mainly delivered one grasshopper or one mantis per nest visit, and these prey items were usually not split between nestlings (W.G., personal observation). Also, grasshoppers are limited in calcium [40], which represents a critical nutrient for avian growth [41,42], thus potentially limiting the growth of black coucal nestlings compared with white-browed coucal nestlings. Hence, white-browed coucals visited their nests less frequently than black coucals, but they may have compensated by delivering larger, calcium-rich and possibly divisible food items.

### 4.1. Microhabitat use and differences in parental care

Territories of black and white-browed coucals often overlap [9], but the two species differ in microhabitat use. For example, white-browed coucals typically nest in acacia shrubs or bushes close to water bodies and typically forage in open areas with scattered vegetation. By contrast, black coucals nest and forage in dense and high grasses ([9] and personal observation of all authors). Also, there is little evidence for interspecific competition between the two species that rarely interact, except when they compete for favourite resting perches. This suggests that they may occupy slightly different ecological niches, which are reflected by the differences in nesting and foraging microhabitats, and the type of food delivered to the nestlings.

We consider it unlikely that both parents are needed to successfully raise a brood in white-browed coucals: first, despite the differences in microhabitat use, both species experience the high food abundance typical for the Usangu during the rainy season. Resource levels are so high that in black coucals a single parent is sufficient to successfully raise a brood. Second, the feeding rates of a single white-browed coucal amounted to less than one-third, and the combined feeding efforts of both parents added-up to only about 50% of the performance of a single-parenting male black coucal. We consider it unlikely that these differences in feeding rates were the result of differences in foraging efficiency, because between two feeding visits white-browed coucals typically spent considerable time perching high on bushes or grasses, where they preened or warmed-up in the sun without searching for food. Male black coucals had much less of such ‘off-times’ between feeding visits. Hence, it is unlikely that white-browed coucals needed more time to search for food than black coucals. Further, the feeding rates of white-browed coucals were extremely low compared with the few data available from other biparental coucal species, e.g. the pheasant coucal (*Centropus phasianinus*) with 3.8 feedings per hour [18] which corresponds to the average feeding rate of a uniparental male black coucal in this study (see also [24]).

The lower overall feeding rate of biparental white-browed coucals compared to uniparental black coucals is consistent with mathematical models of sexual conflict in offspring care: in biparental species, sexual conflict over care should lead to suboptimal contributions of each individual parent to prevent exploitation by the other parent (e.g. [43,44]). Experimental data in captive zebra finches [45,46] and canaries [47] support these models. Hence, one reason why male black coucals work harder than white-browed coucals could be the absence of a sexual conflict over provisioning the young in a species in which only one sex cares. Given the similarity in brood sizes, the expected net workload should be twice as high for a male black coucal compared with the individual members of a pair of white-browed coucals. The fact that male black coucals work harder than that is consistent with theoretical expectations regarding the absence of a sexual conflict over care.

### 4.2. Parental care, parental effects and mating system

The sparse literature on coucals indicates that in most species both parents contribute to parental care, possibly with a male-bias in incubation [17]. The only exception is the black coucal in which only males care [23,48]. Hence, biparental care most probably represents an ancestral trait in coucals. We showed that even in white-browed coucals—probably representing the coucal species with the highest similarity in sex roles—males take a larger share in incubation than females, at least during the day. Andersson [13] suggested that the involvement of males in incubation was a necessary starting point for sex-role reversal and polyandry in black coucals and provided evidence that females of many socially monogamous coucal species have intervals of 2–3 or even more days between laying two eggs. These long intervals may indicate a difficulty to gather sufficient resources for rapidly finishing a clutch. Thus, females of all coucal species could benefit from male assistance during incubation, because it frees them from the costs of incubation and they gain additional time to gather resources for egg-laying.

Limitation of food is a rather unlikely factor for the reproduction of coucals in the Usangu plains. Thus, differences in food abundance, food accessibility, foraging efficiency or a lower need for parental care are unlikely causes for the differences in parental care between black and white-browed coucals. In contrast to our predictions, offspring feeding rates were substantially lower in pairs of white-browed coucals than in uniparental male black coucals, but these differences did not result in differences in offspring growth. Hence, a good food supply may be a necessary factor for the evolution of uniparental care [13,49,50], but does not inevitably lead to uniparental care even in closely related and ecologically similar species. Given their low feeding effort, we consider it unlikely that two white-browed coucal parents are required to successfully raise a clutch. Rather, a lower breeding density and a more balanced adult sex-ratio may prevent both sexes from finding additional mating partners [9]. Some shorebirds simultaneously raise multiple clutches with the male incubating the first clutch and the female incubating the second clutch (e.g. Temminck's stilt *Calidris temminckii* [51]). What prevented female white-browed coucals from evolving a similar strategy? This remains an open question, but it could be related to the longer breeding season with the possibility to raise several clutches as a pair in succession (up to four successful broods per season, W.G. and I.S., unpublished data). As a consequence, the reproductive value of a single brood (and also a single breeding season) may be lower, and the value of a prolonged pair-bond may be higher in white-browed coucals than in migratory shorebirds that experience very short breeding seasons.

Black coucals, on the other hand, with their higher breeding densities and a strongly male skewed adult sex-ratio [9] render themselves much more likely for male-only care and polyandry [6,7]. However, males may pay a physiological price for their higher feeding effort, because feeding male black coucals express higher levels of baseline and stress-induced corticosterone concentrations than non-feeding males. By contrast, feeding individuals of white-browed coucals do not show such elevations in baseline and stress-induced corticosterone [52]. Whether these physiological differences affect survival is unknown.

In a previous study, we have identified several factors that may have represented the initial steps on the black coucal's route to obligate sex-role reversal and polyandry [9]. Being the only coucal species that migrates, black coucals can gather in breeding habitats with temporarily high food resources and breed at high densities, which facilitates the monopolization of more than one breeding partner. Because high egg-predation in black coucals selects for females that rapidly produce replacement clutches and for males that fully take over incubation, female emancipation from incubation created a new sexual selection pressure for females to compete for additional mates. If the clutch incubated by her first mate is successful, the female is free to search for additional mates. Sexual selection may then have favoured large females that can compete over territories and mates [53,54] and that lay a large number of eggs [9]. At the same time, selection may have favoured small body size in males, because small males make more efficient foragers to raise the young [27]. This development probably generated several positive feedback loops on sexual size dimorphism, with the potential consequence of higher female juvenile mortality resulting in a male-biased adult sex-ratio [9], which according to recent theory should enforce competition in females and male-only care [5–7]. These selective forces may have resulted in the complete and invariable sex-role reversal observed in all black coucal populations investigated so far [23,48,55].

By contrast, nest predation in white-browed coucals is considerably lower [9] and hence, female white-browed coucals may benefit less from being large and liberated from incubation. Thus, there may be little selection for sexual size dimorphism and white-browed coucals lack the morphological, physiological and behavioural adaptations for female competition and male-only care observed in black coucals [24,53,54,56–58]. These findings support theoretical models of sex roles in mating and parental care [5–7] and recently published meta-analyses in birds [8,59]. Thus, we conclude that white-browed coucals of the Usangu plain are likely to maintain a socially monogamous mating system with biparental care not because two parents would be needed to successfully raise the offspring, but rather because females (or males) may lack opportunities to find additional mates. If the adult sex-ratio of white-browed coucals in the Usangu would be biased towards the one or other sex, or if predation in the egg stage would be higher, we predict that this species would have the potential for developing a polygamous mating system.

In summary, in the Usangu habitat—a flood plain with rich food resources for coucals during the breeding season—biparental white-browed coucals provision their offspring less frequently than uniparental black coucals. Biparental care is considered an ancestral trait in coucals and superabundant food resources have been considered a key factor in the evolution of female emancipation from parental care, and sex-role reversal in black coucals and other species with a similar mating system [13,50]. Our study indicated that rich food resources are a necessary, but not a sufficient condition for a reversal in sex roles and uniparental care. Compared to black coucals, offspring provisioning rates were low in white-browed coucals and a single parent would most probably have been sufficient to raise a clutch. However, white-browed coucals have a relatively balanced adult sex-ratio, reducing the opportunities for either sex to find additional mating partners. Further, white-browed coucals experienced a longer breeding season and lower nest predation rate compared to black coucals, thus reducing the potential benefits of male-only incubation [9]. As a consequence, biparental care may be the best option for both partners, thus balancing also the operational sex-ratio. Our case study confirms a recent comparative dataset in birds showing that the degree of parental cooperation in offspring care is determined by the magnitude of sexual selection and the bias in the adult sex-ratio [10]. But our study also highlights the importance of facilitating factors including life history (migration/breeding density) in combination with high food abundance and high nest predation in the evolution of male-only care in black coucals. A detailed comparison of the degree of sexual selection and the risk of offspring predation in taxa with species where males contribute more to offspring care than females (mainly amphibians and birds) and experimental manipulation of adult sex-ratios or nest predation rates could shed additional light on ecological factors that influence parental care and sex roles in terrestrial vertebrates.

## Supplementary Material

Feeding rate analysis Alternative feeding rate analysis using the number of nestlings and the mean age of nestlings instead of brood body mass Growth rates based on tarsus lengths Supplementary growth rate analyses based on the growth of the right tarsus of coucals instead of body mass data

## Data accessibility

The data used for the analyses are available on Dryad: http://dx.doi.org/10.5061/dryad.23gt0 [60].
