# Supplementary material for: Sex roles, parental care and offspring growth in two contrasting coucal species
Source: R Soc Open Sci. 2016 Oct 5;3(10):160463. doi: 10.1098/rsos.160463 (PMC5098986; doi:10.1098/rsos.160463)
Supplement: Feeding rate analysis Alternative feeding rate analysis using the number of nestlings and the mean age of nestlings instead of brood body mass Growth rates based on tarsus lengths Supplementary growth rate analyses based on the growth of the right tarsus of coucals instead of body mass data [file rsos160463supp1.pdf]

Supplementary analyses

Supplement 1: Feeding rate analysis using number of nestlings and mean nestling age instead of brood mass as covariates

We repeated the feeding rate analysis using the number of nestlings and mean nestling age instead of brood mass as a covariate. The results of this analysis were very similar to the one presented in the main text of the manuscript. The offspring feeding rates differed between the two species and sexes (species x sex interaction: F=88.02, species: F=227.13, sex: F=10.89). The mean [± 95% credible intervals] feeding rate of male black coucals was 2.93 [2.64 – 3.26], of female black coucals was 0.009 [0.003 – 0.030], of male white-browed coucals was 0.79 [0.66 – 0.95], and of female white-browed coucals was 0.67 [0.55 – 0.80] feeding visits per hour. In both species the feeding rate increased with the number of nestlings (slope 0.339 [0.257 – 0.419]; F=64.03) and with the mean age of the nestlings (slope 0.157 [0.095 – 0.220]; F=24.01), but the time of day did not have a meaningful impact (-0.014 [-0.070 – 0.042]; F=0.208). The overall model explained a large proportion of the variance in the data (marginal R^2=0.828, conditional R^2=0.843).

Supplement 2: Tarsus growth rates of white-browed coucal and black coucal nestlings

On the species level, the growth constants K and their 95% confidence intervals indicated that the tarsi of black and white-browed coucals grew at slightly different rates (black coucals faster than white-browed coucals), but with little differences between the sexes (Table S1). Similar to the situation in body mass, male black coucals reached the period of maximum tarsus growth (inflection point I) earlier than females and also earlier than white-browed coucals (Table S1). The asymptotic tarsus length just before the young left the nest also differed between the species and the sexes. Female black coucals reached a larger asymptotic tarsus length than males, and both male and female black coucals left the nest with a shorter asymptotic tarsus length than mass than male and female white-browed coucals (Table S1).

Upon leaving the nest, female black coucals reached 81.1 % of the mean adult tarsus length (42.4 mm) and male black coucals reached 84.3 % of the mean adult tarsus length (38.9 mm). Female white browed coucals reached 87.4 % of the mean adult tarsus length (42.0 mm) and males 87.1 % (adult: 41.1 mm; tarsus lengths of adult coucals were taken from [9]). Thus, also with respect to structural growth female black coucals lagged behind males and behind female and male white-browed coucals.

Table S1: Mean estimates and 95% confidence intervals for the growth constant (K), the inflection point (I) and the asymptotic tarsus length (in mm) (A) before leaving the nest for white-browed and black coucals.

Species	sex	growth constant (K)	inflection point I (day)	asympt. tarsus length A (mm)
white-browed coucal	female	0.207 [0.199; 0.215]<br>t_{461}=52.03	6.3 [5.9; 6.7]<br>t_{461}=31.04	36.7 [35.2; 38.1]<br>t_{461}=50.18
	male	0.217[0.209; 0.225]<br>t_{484}=55.90	6.0 [5.7; 6.3]<br>t_{484}=37.65	35.8 [34.8; 36.8]<br>t_{484}=71.66
black coucal	female	0.225 [0.217; 0.233]<br>t_{484}=55.51	6.1 [5.8; 6.5]<br>t_{484}=37.84	34.4 [33.3; 35.5]<br>t_{484}=61.55
	male	0.228 [0.218; 0.239]<br>t_{499}=42.25	5.6 [5.2; 6.0]<br>t_{499}=28.65	32.8 [31.6; 34.05]<br>t_{499}=51.99

On the level of individual nests there were consistent correlations between the asymptotic tarsus length and the inflection point in male black coucals and female white-browed coucals: in nests where nestlings reached the inflection point earlier the nestlings also reached a higher asymptotic tarsus length (as indicated by the fact that the 95% confidence intervals of the correlation coefficient did not include zero; Table S2). In female black coucals and male white-browed coucals this was not the case, because the 95% confidence intervals of the correlation coefficients included zero (Table S2). On the level of individual nestlings, inflection points and asymptotic tarsus lengths showed consistent correlations in female white-browed and female black coucals, but not in males of the two species (Table S2, right column). Overall, individual nests and individual nestlings varied in asymptotic tarsus lengths and inflection points, as indicated in the estimated random effects standard deviations and their respective 95% confidence intervals (Table S2), suggesting that consistent individual differences on the nest and nestling level play a role in growth parameter estimation.

Table S2: Estimates and their 95% confidence intervals for the standard deviations of random effects of asymptotic body mass before leaving the nest (A) and the inflection point (I), and their respective correlations (with 95% confidence intervals) on the nest (left) and the nestling level (right). Biologically meaningful correlation coefficients are highlighted in bold.

Species	sex	nest ID			nestling ID		
		asymptote A	infl. point I	correlation	asymptote A	infl. point I	correlation
black coucal	fem	0.7[0.1;3.9]	0.7 [0.5;1.0]	0.9 [-1;1]	3.3 [2.7;4.0]	0.4 [0.2;0.7]	**0.6 [0.1;0.9]**
	male	2.0 [1.2;3.4]	0.8 [0.6;1.1]	**0.6 [0.1;0.9]**	2.5 [1.8;3.4]	0.6 [0.3;0.9]	0.4 [-0.1;0.7]
white-browed coucal	fem	2.7 [1.8; 4.2]	0.9 [0.6; 1.2]	**0.6 [0.2; 0.9]**	3.4 [2.6; 4.3]	0.7 [0.4; 1.0]	**0.6 [0.3; 0.8]**
	male	1.6 [1.0; 2.7].	0.7 [0.5; 1.0]	0.4 [-0.2; 0.7]	2.0 [1.4; 2.7]	0.4 [0.2; 0.7]	0.2 [-0.4; 0.7]
